# Stereotactic body radiotherapy for early-stage lung cancer: a systematic review on the choice of photon energy and linac flattened/unflattened beams

**DOI:** 10.1186/s13014-023-02392-4

**Published:** 2024-01-02

**Authors:** Ashlesha Gill, Andrew L Hirst, Pejman Rowshanfarzad, Suki Gill, Nicholas Bucknell, Joshua Dass, Mahsheed Sabet

**Affiliations:** 1 School of Physics, Mathematics and Computing, The University of Western Australia, Crawley, WA 6009 Australia; 2 Department of Radiation Oncology, Sir Charles Gairdner Hospital, Nedlands, WA 6009 Australia; 3 Sir Peter MacCallum Department of Oncology, University of Melbourne, Melbourne, VIC 3000 Australia

**Keywords:** SBRT, SABR, Lung cancer, Radiotherapy, Photon energy

## Abstract

SBRT is an effective local treatment for patients with early-stage non-small cell lung cancer (NSCLC). This treatment is currently used in patients who have poor lung function or who decline surgery. As SBRT usually has small PTV margins, reducing the beam-on-time (BOT) is beneficial for accurate dose delivery by minimising intrafraction motion as well as improved patient comfort. Removal of the linear accelerator flattening filter can provide a higher dose rate which results in a faster treatment. In addition, the choice of photon energy can also affect the dose distribution to the target and the organs-at-risk (OAR). In this systematic review, studies analysing the choice of various photon beam energies, with a flattening filter or flattening filter free (FFF), were compared for their overall dosimetric benefit in the SBRT treatment for early-stage NSCLC. It was found that FFF treatment delivers a comparatively more conformal dose distribution, as well as a better homogeneity index and conformity index, and typically reduces BOT by between 30 and 50%. The trade-off may be a minor increase in monitor units for FFF treatment found in some studies but not others. Target conformity and OAR sparing, particularly lung doses appear better with 6MV FFF, but 10MV FFF was marginally more advantageous for skin sparing and BOT reduction. The favourable beam modality for clinical use would depend on the individual case, for which tumour size and depth, radiotherapy technique, as well as fractionation scheme need to be taken into account.

## Background

Stereotactic Body Radiation Therapy (SBRT) emerged from stereotactic radiosurgery (SRS), where successful treatments of brain tumours were delivered using a single high dose fraction with a steep dose falloff [1–4]. Therefore, the earlier definition of SBRT focussed on using a stereotactic body frame for patient setup, which allowed conformal dose distribution to be delivered to an extracranial target in a hypo-fractionated treatment scheme [5]. Now, image guidance techniques are preferred and can provide a frameless method for patient setup. Current definitions by various organisations indicate that SBRT is a form of external beam radiotherapy, in which a high dose of radiation is accurately delivered to an extracranial target in a small number of fractions [6–9].

SBRT is commonly used to treat primary cancers of the lung, prostate, kidney, liver, and pancreas [10] and oligometastatic cancers in the lung, liver, lymph nodes, adrenal gland, and spine [11]. This review focuses on the treatment of early-stage non-small cell lung cancer (NSCLC) using SBRT. Global data from 2020 shows that lung cancer has been the second most commonly diagnosed cancer and responsible for the highest number of cancer deaths [12]. NSCLC comprises 85% of lung cancer diagnoses with about 15% at a localised early stage [13, 14], for which conventionally, surgical resection has been the standard treatment [15]. However, patients may be deemed medically inoperable, due to a high possibility of treatment complications associated primarily with poor pulmonary function, or other factors such as performance status, cardiovascular disease, age and comorbidities. SBRT has replaced conventional radiotherapy as the standard treatment for inoperable early-stage NSCLC patients [16]. Local control (LC) rates for 3- and 5- year follow up periods range between 78 − 98% and 79 − 85%, respectively for early-stage NSCLC when treated with SBRT [14]. This is a significant improvement compared to LC rates for treatment with conventional radiotherapy, which for 2- and 5- year follow up periods, were typically around 40% and 10%, respectively [16].

Considerable research on factors affecting patient outcomes for SBRT of the lung have been accumulated over the past few decades, including image-guided radiotherapy (IGRT) and motion management [17], patient conditions [18], fractionation schedules [15] and tumour size [15]. Due to the complex nature of SBRT, the required immobilization, verification, and treatment delivery lead to considerably longer treatment times [19, 20]. In addition to patient discomfort, longer treatment times have the potential to increase intrafraction patient motion, which would be unsuitable for the required tight margins for SBRT treatments [21]. The speed of Volumetric Modulated Arc Therapy (VMAT) delivery is generally limited by the maximum dose rate imposed by the flattening filter. While removal of the flattening filter increases dose rate and decreases treatment time, it results in a non-uniform dose profile [20]. Intensity modulation is used to compensate for this; however, it raises the question: Is the dose distribution of modulated FFF beams equivalent to that of flattened beams?

The key is to achieve a good therapeutic ratio along with minimal intrafractional motion, which is especially important in the given case as the treatment involves very high doses delivered in a few fractions. This review aims to investigate dosimetry for photons beams with or without flattening filter, as well as various photon energies, to establish the optimal choice for lung SBRT, particularly in terms of factors such as target coverage, sparing of critical organs, treatment delivery time and the number of monitor units (MUs) used.

## Main text

### Methods

For this review, a literature search was conducted for journals based on PRISMA guidelines (RRID:SCR_018721) within the time period from 2013 to 2023. 5 July 2023 was taken as the cut-off date. The inclusion criteria were: (i) Articles focusing on optimal energies of photon beams for lung SBRT based on dosimetric evaluation. (ii) Articles focusing on comparison of flattened/unflattened photon beams for lung SBRT based on dosimetric evaluation.

The following search string was used to search three databases (Scopus, Web of Science and PubMed):

(“lung” OR “NSCLC”) AND (“SBRT” OR “SABR” OR “stereotactic”) AND (“choice of energy” OR “energy choice” OR “optimal energy” OR “best energy” OR “*MV " OR “*FFF” OR “with flattening filter” OR “without flattening filter” OR “flattening filter free” OR “flattened” OR “unflattened”).

**Fig. 1:**
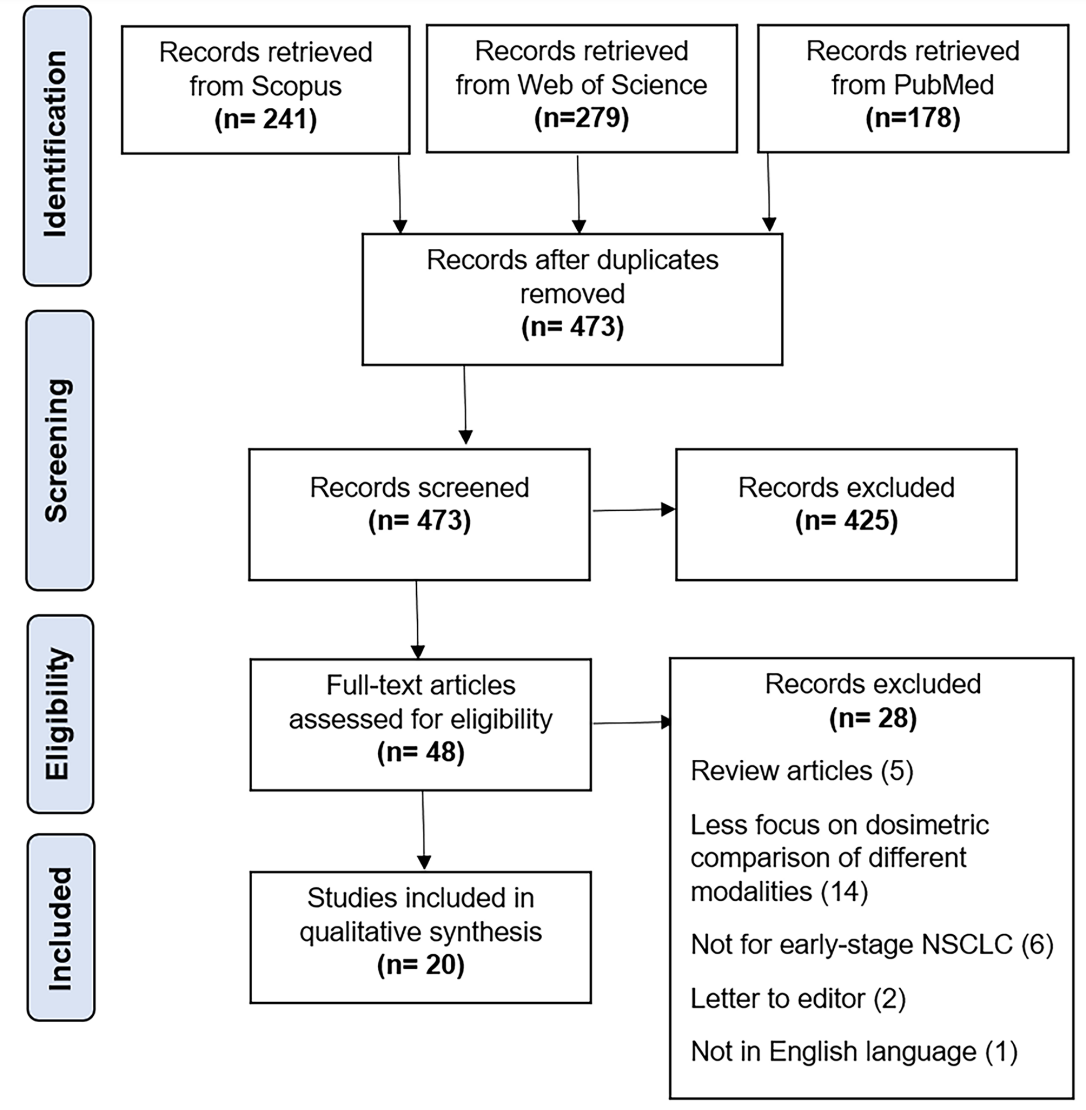
PRISMA flow diagram showing the literature review process

Studies were first screened by title and abstract and duplicates or those that didn’t meet the inclusion criteria were excluded (Fig. 1). 48 full-text articles were then retrieved for review, out of which 28 articles were excluded based on the reasons detailed in Fig. 1. This resulted in a total of 20 studies included in this review (Table 1).

**Table 1:** Primary study characteristics and results

	Beam modalities	Number of patients	Optimisation algorithm	Fractionation schemes	Favourable beam modality as per the specified criteria
Hrbacek et al. 2014 [32]	6-FF, 6-FFF, 10-FFF	11	AAA	50 Gy/5 fx	6-FFF (Target conformity) 10-FFF (BOT reduction, lower skin and peripheral dose)
Lu et al. 2015 [36]	6-FFF, 10-FFF	12	AAA	48 Gy/4 fx, 54 Gy/3 fx, 34 Gy/ 1 fx	6-FFF (For 48 Gy/4 fx and 54 Gy/3 fx) 10-FFF (For 34 Gy/1 fx)
Kim et al. 2015 [27]	6-FF, 6-FFF	10	AcurosXB	48 Gy/4 fx	6-FFF (BOT reduction)
Zhang et al. 2015 [46]	6 MV, 3 MV, mixed energy of 3 and 6 MV	31	Monte Carlo	50 Gy/4 fx, 54 Gy/3 fx, 40 Gy/4 fx, 50 Gy/5 fx	3 MV (Target conformity, homogeneity, and OAR sparing for physically thin patients)
Liu et al. 2016 [24]	Flattened 6 MV, 6-FFF	98	AcurosXB	48 Gy/4 fx	6-FFF (BOT reduction)
Barbiero et al. 2016 [23]	Flattened 6 MV, 6-FFF	25	AAA	24 Gy/1 fx	6-FFF (BOT reduction)
Vieillevigne et al. 2016 [29]	Flattened 6 MV, Flattened 10 MV, 6-FFF, 10-FFF	24	AAA	60 Gy/3 fx	6-FFF, 10-FFF (Target conformity, BOT reduction, except in the case of DCA treatment of larger tumour)
Tambe et al. 2016 [34]	Flattened 6 MV, 6-FFF, 10-FFF	15	AcurosXB	18 Gy/fx, 11 Gy/fx, 7.5 Gy/fx, 6.25 Gy/fx	10-FFF (BOT reduction)
Aoki et al. 2017 [22]	Flattened 6 MV, Flattened 10 MV, 6-FFF, 10-FFF	30	Collapsed Cone	55 Gy/4 fx	6-FFF, 10-FFF (BOT reduction)
Pokhrel et al. 2019 [28]	Flattened 6 MV, 6-FFF	13	AcurosXB	30 Gy/1 fx	6-FFF (OAR sparing, BOT reduction)
Vassiliev et al. 2020 [26]	Flattened 6 MV, 6-FFF	15	AAA	50 Gy/4 fx, 63 Gy/7 fx, 70 Gy/7 fx,	6-FFF (Target coverage, OAR sparing)
Akbari et al. 2021 [33]	6-FFF,10-FFF	73	Collapsed Cone	50 Gy/5 fx, 60 Gy/5 fx	6-FFF (Target homogeneity, dose fall-off)
Wei et al. 2022 [31]	6 MV, 10 MV	30	Collapsed Cone	48 Gy/4 fx	6 MV (OAR sparing)
Savanovic et al. 2022 [25]	Flattened 6 MV, 6-FFF	100	Collapsed Cone	60 Gy/4 fx, 60 Gy/8 fx	6-FFF (OAR sparing, BOT reduction)
Gasic et al. 2014 [30]	Flattened 6 MV, Flattened 10 MV, 6-FFF, 10-FFF	20	AAA	-	6-FFF, 10-FFF (BOT reduction) 6-FF, 10-FF (Target homogeneity in larger tumours)
Wu et al. 2023 [35]	Flattened 6 MV, 6-FFF	198	Collapsed Cone	50 Gy/5 fx	6-FFF (OAR sparing, BOT reduction)
Gaudreault et al. 2022 [37]	FF, FFF	366	-	18–28 Gy/1 fx	FFF (BOT reduction)
Nielsen et al. 2016 [38]	FF, FFF	745	Collapsed Cone	45 Gy/3 fx	FFF (BOT reduction)
Pokhrel et al. 2019 [39]	Flattened 6 MV, 6-FFF	13	AcurosXB	30 Gy/1 fx	6-FFF (BOT reduction)
Miura et al. 2020 [40]	6-FFF, 10-FFF	10	Collapsed Cone	42 Gy/4 fx	6-FFF (OAR sparing) 10-FFF (BOT reduction)

## Results

### Target coverage

Some studies conclude that flattened and FFF plans provide similar PTV/ITV coverage [22–25]. Results from a study by Vassiliev et al. [26]. indicate that FFF plans provide better target coverage by improving D98, D95, D90, homogeneity index and uncomplicated tumour control probability for the PTV. Kim et al. [27]. compared the target volume DVHs for 6MV plans with flattened and FFF beams and observed only a small difference. The dose was adequate for target coverage in all the plans. Both, Aoki et al. [22]. and Liu et al. [23]. mentioned that their flattened and FFF plans achieved similar conformity and homogeneity. However, Kim et al. [27]. found that FFF beams provided comparatively more conformal dose distribution and provided more homogeneous plans. Savanovic et al. [25]. have reported that the conformity index improved by 7% when using FFF beams. This was in agreement with the findings of Pokhrel et al. [28]., who noted that the 6-FFF beam improved conformity with less intermediate-dose spillage and smaller hot spots compared to flattened 6 MV beams. Additionally, the benefit was more in the case of island tumours which are surrounded by ipsilateral low lung density. Vieillevigne et al. [29]. and Gasic et al. [30]. mentioned that as the PTV volume increases, FFF beam tends to be less beneficial in terms of homogeneity. This effect is observed to be significant for PTV volumes greater than 43.97 cm^3^ and more so for the dynamic conformal arc (DCA) technique [29].

Wei et al. [31]. compared 6 MV and 10 MV plans and reported that there wasn’t a major difference between them in terms of conformity index, heterogeneity index, and D2%. Akbari et al. [33] indicate that 6-FFF provides better plan homogeneity, for instance in the case of small-size lesions, homogeneity index (HI) for FFF plans is 0.32 with 6 MV and 0.34 with 10 MV and in the case of large-size lesions, HI is 0.27 with 6 MV and 0.38 with 10 MV. Other studies comparing 6 MV flattened and FFF beams and 10 FFF beams report that that 6 FFF is also the most optimal in terms of conformity for the dose distribution [32], sharper dose fall off [33, 34], and target coverage [34].

### OAR sparing

There was no statistically significant difference in dose to OARs for flattened and FFF techniques when used for VMAT-SBRT, as reported by Hrbacek et al. [32]., Aoki et al. [22]., Barbiero et al. [23]. and Kim et al. [27]. The dose to OARs including ribs, lungs, and skin was lower for 6-FFF, in comparison to flattened 6 MV beams, for VMAT planning as shown by Pokhrel et al. [28]. FFF beams can significantly lower the mean lung dose, V10, V20, V30, and normal tissue complication probability (NTCP) for the total lung as well as the ipsilateral lung [26]. Savanovic et al. [25]. pointed out that, irrespective of the tumor location (central or peripheral lung) or the technique used, which was either dynamic conformal arc (DCA) or static field (SF), 6-FFF reduced the dose to the OARs. The results from Vieillevigne et al. [29]. comparing flattened 6 MV plans with 6-FFF and flattened 10 MV plans with 10-FFF, confirmed that FFF beams provided better organ sparing than flattened beams. However, 10-FFF was shown to be less beneficial in the case when it is used with DCA for medium and large targets [29]. Wu et al. [35]. compared the two techniques for target sizes in the range of 3.8 cm^3^ to 34 cm^3^ and suggested that the improvement in the critical organs’ sparing with the use of FFF beams increased with increasing target volume. The difference in skin sparing between flattened and FFF beams was not statistically significant [28, 34].

Lu et al. [36]. compared 6-FFF with 10-FFF, and showed that 6-FFF beam significantly lowered the dose to all OARs owing to its sharper dose penumbra. Consequently, the NTCP was reduced by 10% for both the lung and chest wall, in the fractionation schemes of 4 × 12 Gy and 3 × 18 Gy. However, for 1 × 34 Gy, the reduction was 7.4% for lung and 2.6% for chest wall. In the case of heart, oesophagus, and spinal cord, NTCP reduction was negligible. This was similar to the findings of Wei et al. [31]., whose study demonstrated that 6 MV plans resulted in 4.68–8.91% lower D_max_ for spinal cord, oesophagus, great vessels, and trachea and proximal bronchial tree than 10 MV plans. Also, the D_mean_, V5Gy, V10Gy, and V20Gy of whole lung were reduced by 2.79–5.25% using 6 MV beams. Tambe et al. [34]. compared flattened 6 MV, 6-FFF and 10-FFF plans and showed that OAR sparing improved by using 6-FFF. Although statistically significant, the absolute dose differences were not clinically significant. The doses to OARs were below the recommended tolerance limits for each case. As expected, 10-FFF resulted in better sparing of the skin compared to 6-FFF.

### BOT reduction and MUs

Studies have shown that the beam-on time (BOT) was reduced by a factor of 2.3 when 6-FFF beam was used, compared to flattened 6 MV beams [22, 23, 28]. This was in agreement with results from Kim et al. [27]., who reported a 52.97% reduction in treatment delivery time with the use of 6-FFF beams. Savanovic et al. [25]. investigated this for different radiotherapy techniques, and stated that the median gain achieved with beam-on time with FFF beams ranged from 31 to 34% for static field technique and 44–52% for DCA. The use of FFF beams reduced the treatment time by 28% as shown by Gaudreault et al. [37]., where most treatments were performed using 3D-CRT. Nielsen et al. [38]. mentioned that lung SBRT with FFF beams reduced the treatment time by 21% and the intrafractional motion was reduced from 1.9 mm to 1.6 mm. Treatments could be delivered in a 15 min. slot using 6-FFF VMAT [39]. Regarding the MU values, Aoki et al. [22]., Barbiero et al. [23]., and Pokhrel et al. [28]. reported no significant different between flattened and FFF beams whereas other studies showed that FFF beams required larger number of MUs. For example, Kim et al. [27]. reported that FFF beams required 4.65% more MUs and Liu et al. [24]. reported that for VMAT, FFF beams required 6.7% more MUs.

While comparing 6-FFF, 10-FFF and flattened 6 MV beams, many studies have indicated that both FFF beams significantly reduced BOT, also compensating for the increase in MUs [29, 32, 34]. Tambe et al. [34]. highlighted that 6-FFF and 10-FFF reduced the treatment delivery time by 2.2 min (55%) and 2.5 min (61%) respectively, compared to flattened 6 MV beams. It was observed that 10-FFF provided slightly better treatment efficiency compared to 6-FFF. Results from Hrbacek et al. [32]. and Miura et al. [40]. support this as well. Miura et al. [40]. suggested using 10-FFF applied under a robust optimized plan for treatments with breath-hold technique. Lu et al. [36]. compared 6-FFF and 10-FFF for various fractionation schemes and found that 10-FFF reduced beam-on time by 31.9%, 38.7% and 43.6% for 4 × 12, 3 × 18 and 1 × 34 Gy schemes, respectively.

## Discussion

In SBRT treatments, it is essential to maintain an accurate treatment position as higher doses are delivered in fewer fractions leaving less leeway for error. The BOT needs to be reduced to minimise the intrafractional variation, along with ensuring a good therapeutic ratio.

Lung SBRT is associated with high tumour control rates for both single-fraction [41] and multifraction treatments [41–43]. In the case of single-fraction SBRT, flattened 6 MV and 6-FFF VMAT resulted in equivalent dosimetric parameters for PTV and OARs; and BOT was significantly reduced with the use of 6-FFF beams as expected [23]. However, Pokhrel et al. [28, 39]. suggested that 6-FFF improved target conformity, dose coverage at tumour interface and OAR sparing. This is more prominent for lower ipsilateral lung density [28]. Lu et al. [36]. recommended using 6-FFF for 3 × 18 Gy and 4 × 12 Gy schemes, and 10-FFF for 1 × 34 Gy scheme. The tumour reportedly remains in a considerably stable position if the treatment delivery time is 6 min or less [44, 45]. This implies that tumour motion is not the primary concern in the case of 3 × 18 Gy and 4 × 12 Gy schemes (BOT in the range 1.5-4 min), and they would benefit the most from 6-FFF beams, which provides better OAR sparing. The BOT for 1 × 34 Gy scheme was 6.3 min with 6-FFF, and 3.5 min with 10-FFF, indicating that 10-FFF would be beneficial for this fractionation scheme.

The flattening-filter free technique can be used without compromising the target coverage [22–26]. Due to the lower beam energy of FFF beams, there is less scattering in linear accelerator head, and less MLC leakage and scattering in the medium. As a result, there is a sharp dose gradient outside the PTV. This can be beneficial in improving the beam conformity as well as reducing the OAR dose [25, 27, 28]. FFF beams can considerably lower the NTCP for critical organs [26, 36]. Although FFF plans are advantageous for OAR sparing, it is difficult to achieve such good results in the case of larger tumours [26]. Vieillevigne et al. [29]. compared DCA or VMAT plans using various flattened and FFF beams, and showed that particularly for DCA, the conformity and healthy-tissue sparing was suboptimal when 10-FFF was used for medium and large targets. Meanwhile, the results from Wu et al. [35]. suggested that the OAR sparing improved with FFF beams for large targets. The studies reviewed in the present article showed that differences in target coverage, CI, HI, and OAR sparing between the flattened and FFF beams are generally small and may not be clinically significant.

There are relatively fewer studies that investigate dosimetry for different energies of photon beams, for example, 6-MV, 10-MV, and intermediate megavoltage beams (< 6-MV). Based on the literature comparing 6-FFF,10-FFF and flattened 6 MV beams, it is indicated that all provide comparable dose distributions to the target [31, 32, 34]; however, 6-FFF can significantly improve conformity and OAR sparing [22, 29, 31, 32, 34]. This is attributed to sharper penumbra at shallow depths and small fields, caused by the shorter secondary particle ranges, which is particularly advantageous for NSCLC treatments [32]. 10-FFF is found to be more beneficial for skin sparing as the maximum dose shifts further away from the surface in comparison to 6 MV beams [34]. It must be noted that in the case of lung SBRT, skin dose is only a concern when the tumour lies very close to the skin. This is a clinically rare situation, and no high-grade skin toxicity has been observed in patients treated with 6-FFF [32]. Zhang et al. [46]. suggested that 3-MV can be a potentially better choice for treatment of patients who are physically thin, as it could further improve the tumour coverage and OAR sparing in comparison to 6-MV beams.

Using FFF beams can reduce the BOT by a factor of 2.3 compared to flattened beams, also compensating for the increase in MUs with FFF [22, 23, 28, 29, 32, 34]. With reduction in treatment time, a subsequent reduction of intrafractional motion is observed, which enables the use of smaller PTV margins [38]. The BOT reduction is slightly greater when 10-FFF is used instead of 6-FFF [32, 34, 36]. Depending on the case examined, the improvement in treatment efficiency can outweigh the small increase in OAR dose [34]. Another factor to consider is the interplay between the target motion and the motion of the photon beam defined by the multi-leaf collimator (MLC) aperture which can affect the accuracy of dose delivery. When the BOT is shorter, the interplay effect becomes a concern and robust optimization may be required to counter it [23]. However, it is practically negligible for treatments using 2-arcs and more than 2 fractions [20].

Of note, type-B dose algorithms, such as AAA (Varian Medical Systems, Inc., RRID:SCR_017372) and Collapsed Cone (Philips, RRID:SCR_008656) have been implemented in various treatment planning studies included in the review instead of type-C dose algorithms, such as Acuros XB (Varian Medical Systems, Inc., RRID:SCR_017372). While comparing AAA with Acuros XB, AAA overestimates the dose quite significantly in the low-density regions of the lung (≤ 0.15 g/cm3) and this effect increases for small tumors (≤ 15-mm diameter) and in the case of 10-MV photon beam [47]. Dose calculations performed with Acuros XB are more accurate than AAA or Collapsed Cone, as well as in good agreement with the X-ray voxel Monte Carlo calculations [48].

It is important to incorporate patient-specific factors in the decision making when selecting the optimal beam modality for the treatment. If the tumour is not at a sufficiently large distance from the skin, 10-FFF would be implemented to avoid skin reactions [34]. The feasibility of the photon beam should be verified with respect to the tumour size [29]. The weight of the patient would also have an impact. As discussed earlier, the use of intermediate MV photon beams might be useful for physically thin patients [46]. In patients with cardiac implantable electronic device (CIEDs), FFF beams with energies greater than 6 MV would not be ideal if the tumour is close to the device [49]. A lower energy, 6 MV, is better suited for patients of older age, with a poorer ECOG performance or a higher Charlson comorbidity index.

Results from previous reviews by Dang et al. [20]. and Ghemis et al. [50] are congruent to the results obtained in the present review, regarding the benefit of FFF-SBRT in reducing the beam-on time along with sufficient tumour control and OAR sparing. The current work extends this analysis to also include comparison of FFF beams with 6 MV or 10 MV energies, providing clinically applicable evaluation of the associated dosimetric effects. The robustness of the results was limited by the lack of literature than compares 6 MV and 10 MV for lung SBRT. Other limitations that need to be addressed are smaller patient group, inadequate use of dose verification tools and few planning studies performed with type-C dose algorithms. Potential investigation of patient outcome with assessment of local control, overall survival, acute and late toxicity, etc. can also be carried out.

## Conclusions

Use of FFF beams facilitates reduction of treatment time, which directly contributes to patient convenience and reduction of workload. The best clinical approach would be to conduct a patient-specific analysis, along with including the effect of radiotherapy technique and fractionation scheme to determine which dosimetric parameters are more critical to optimize in the given case, and the optimal treatment can be selected. Overall, 6-FFF proves to be comparatively advantageous, however in certain instances, 10-FFF may be better where there are significant skin sparing- related or treatment time- related concerns. Understanding of the subject can be consolidated by future studies.

## Data Availability

Not applicable.

## Abbreviations

SBRT: Stereotactic body radiation therapy
NSCLC: Non-small cell lung cancer
BOT: Beam on time
OAR: Organs at risk
FFF: Flattening filter free
PTV: Planning target volume
MV: Megavoltage
SRS: Stereotactic radiosurgery
LC: Local control
IGRT: Image guided radiotherapy
VMAT: Volumetric modulated arc therapy
MU: Monitor units
PRISMA: Preferred Reporting Items for Systematic Reviews and Meta-Analyses
ITV: Internal target volume
DVH: Dose volume histogram
NTCP: Normal tissue complication probability
DCA: Dynamic conformal arc
SF: Static field
3D: CRT 3D-Conformal radiation therapy
MLC: Multi-leaf collimator
AAA: Analytical anisotropic algorithm
CIED: Cardiac implantable electronic device
